# Supplementary material for: Inflammatory Modulation of Polyethylene Glycol-AuNP for Regulation of the Neural Differentiation Capacity of Mesenchymal Stem Cells
Source: Cells. 2021 Oct 22;10(11):2854. doi: 10.3390/cells10112854 (PMC8616252; doi:10.3390/cells10112854)
Supplement: Supplementary file 1 [file cells-10-02854-s001.zip › cells-1381168-supplementary.pdf]

Inflammatory Modulation of Polyethylene Glycol-AuNP for Regulation of the Neural Differentiation Capacity of Mesenchymal Stem Cell

Supplementary data

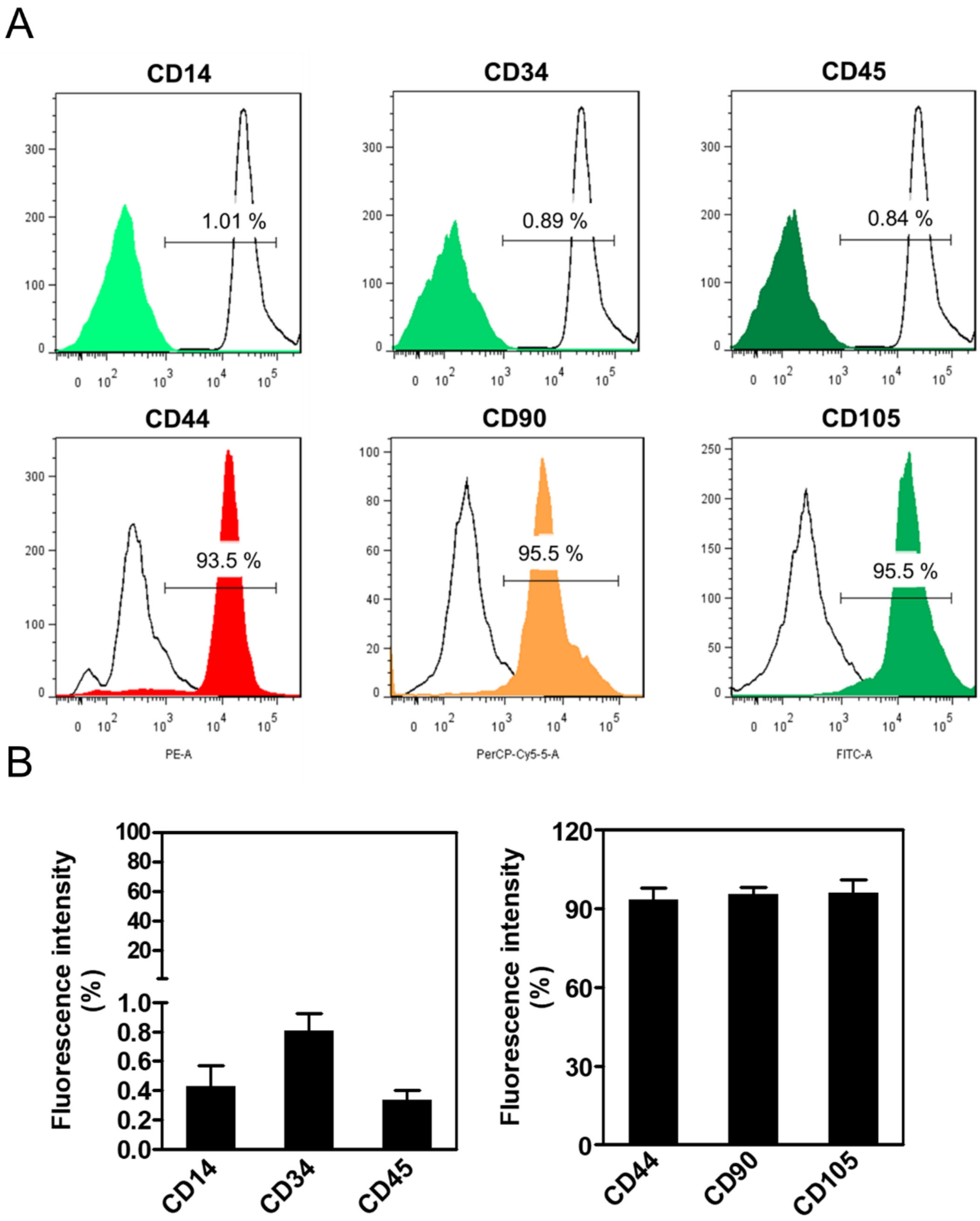

Figure S1. Characterization of the MSCs. **(A)** The specific surface antigens of MSCs were analyzed by flow cytometry. The antibodies were conjugated with fluorescein isothiocyanate (FITC), phycoerythrin (PE) or PerCP-Cy5.5, the following markers were CD14-FITC, CD34-FITC, CD45-FITC, CD44-PE, CD90-PerCP-Cy5.5, and CD105-FITC. **(B)** The left panel demonstrated the quantification of CD14, CD34, and CD45 markers. The right panel indicated the quantitative results of CD44, CD90, and CD105 expression by flow cytometry.

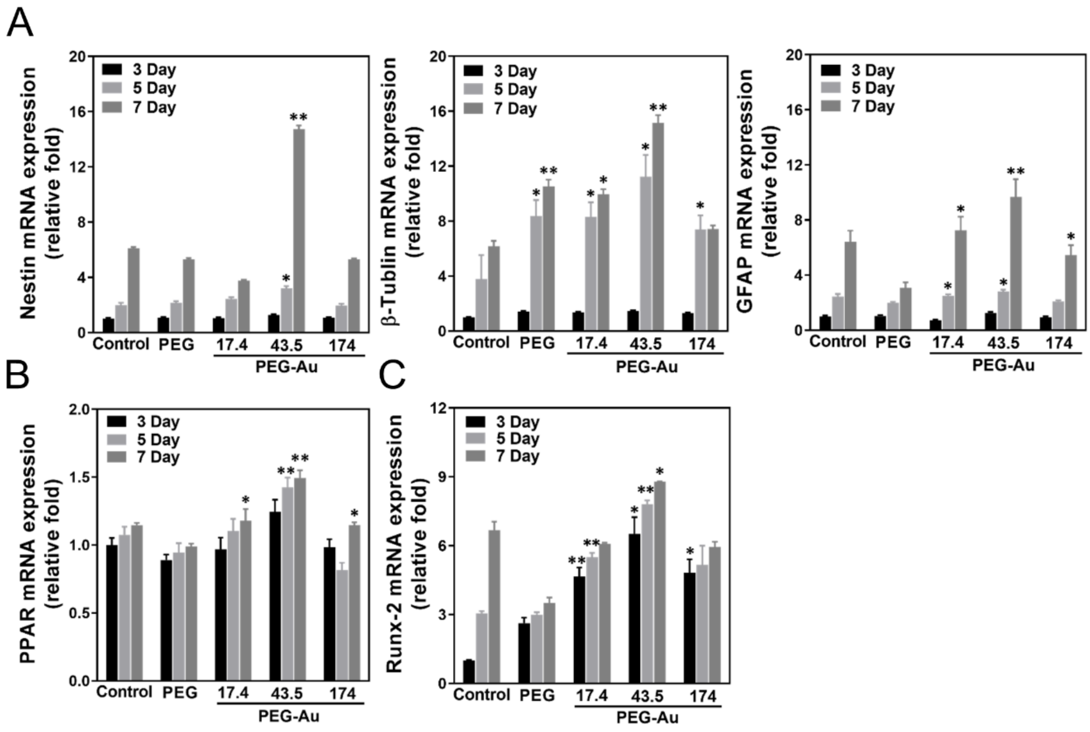

Figure S2. The real time PCR analysis of the mRNA expression level in MSCs after being cultured with various materials. **(A)** The mRNA expression levels of nestin, β-tubulin, and GFAP for neurogenic differentiation. The semi-quantitative results indicate that the expression level of each mRNA was remarkably greater in the PEG-Au 43.5 group at day 7 when compared to the others. **(B)** The mRNA expression level of PPAR for adipogenic differentiation. The semi-quantitative results indicate that PEG-Au 43.5 ppm could significantly induce the mRNA expression at day 7 when compared to the control group. **(C)** The mRNA expression level of Runx-2 for osteogenic differentiation. The semi-quantitative results demonstrate that the expression of Runx-2 was remarkably induced in the PEG-Au 43.5 group at day 7 when compared to the control group. Data are expressed as mean ± SD ($n = 3$). *$p < 0.05$; **$p < 0.01$: greater than the control group.
